# Response to: are there over 200 distinct types of interstitial lung diseases?

**DOI:** 10.1186/s12931-024-02751-z

**Published:** 2024-03-06

**Authors:** Francesco Amati, Anna Stainer, Stefano Aliberti

**Affiliations:** 1 Department of Biomedical Sciences, Humanitas University, Via Rita Levi Montalcini 4, Pieve Emanuele, Milan, 20072 Italy; 2 Respiratory Unit, IRCCS Humanitas Research Hospital, Via Manzoni 56, Rozzano, Milan, 20089 Italy

We’d like to thank Cooley and Fernandez-Perez for their thoughtful comments and feedback on our article. The Authors underline that using standard classification schemes of interstitial lung disease (ILD) clinical diagnoses, the number of unique ILDs is far from exceeding 200. Moreover, the Authors noticed that this number is frequently reported in several papers.

Depending on how you want to slice and dice it, the number of ILDs can vary broadly. Taxonomy requires determining whether to lump together entities in one category or split them apart [1]. The nosology of disease entities has historically relied on clinical and phenotypic features but other information has been progressively incorporated such as imaging, histology, biomarkers, and other data [2]. While phenotypic features may be relevant to the clinical diagnosis of a specific disease, they are not necessarily specific to the underlying cause. This is certainly true in some conditions that are considered to have a Mendelian pattern of inheritance. However, these conditions are rarely recognized in ILD.

It should be emphasized that the classification of ILD has deeply evolved over the last several decades, with increasing attention placed on the multidisciplinary integration of clinical features with radiological and pathological patterns [2]. Moreover, the results of recent trials have sparked the ongoing debate of whether to lump or split ILD patients based on their disease behavior, as in the case of progressive pulmonary fibrosis (PPF) [3]. As suggested by recent data, lumping PPF patients together simplifies ILD management and treatment [4]. On the other hand in the lumping spectrum, some ILDs are almost entirely dominated by lung inflammation. These ILDs are approached much differently in terms of both pharmacological and non-pharmacological interventions. Moreover, splitting specific ILDs into subgroups based on the improving understanding of disease biology has the advantage of distinguishing prognostic trajectories and potentially identifying new targeted therapies [5]. As an example, classifying pulmonary fibrosis (PF) as MUC5B-PF or telomeropathy-PF instead of using the word “idiopathic” might be a better approach to classify the disease based on its behavior [6]. Treating endotypes with targeted therapies based on the expression of specific biomarkers could maximize the effectiveness of existing or new therapies, such as the case of synthetic androgen danazol for patients with short telomeres or the use of N-Acetylcysteine based on TOLLIP gene variants in IPF patients [7, 8]. Thus, the splitting and the lumping approaches are not mutually exclusive. We believe that the ILD field will move to a dynamic disease classification in which treatment approaches will be governed not only by the classification based on etiologies, phenotypes and endotypes but also by the disease behavior [9]. In this context, a treatable traits approach could provide a comprehensive patient-centered precision medicine strategy [9, 10].

## Data Availability

Data sharing is not applicable to this article as no datasets were generated or analyzed during the current study.

## References

[1] Griese M (2022). Etiologic Classification of Diffuse Parenchymal (interstitial) Lung diseases. J Clin Med.

[2] Travis WD, Costabel U, Hansell DM (2013). An official American Thoracic Society/European Respiratory Society statement: update of the international multidisciplinary classification of the idiopathic interstitial pneumonias. Am J Respir Crit Care Med.

[3] George PM, Spagnolo P, Kreuter M (2020). Progressive fibrosing interstitial lung disease: clinical uncertainties, consensus recommendations, and research priorities. Lancet Respir Med.

[4] Flaherty KR, Wells AU, Cottin V (2019). Nintedanib in progressive Fibrosing interstitial lung diseases. N Engl J Med.

[5] Maher TM, Nambiar AM, Wells AU. The role of precision medicine in interstitial lung disease. Eur Respir J Published Online Febr. 2022;3. 10.1183/13993003.02146-2021. 10.1183/13993003.02146-2021 PMC9449482 35115344

[6] Karampitsakos T, Juan-Guardela BM, Tzouvelekis A, Herazo-Maya JD (2023). Precision medicine advances in idiopathic pulmonary fibrosis. EBioMedicine.

[7] Oldham JM, Ma SF, Martinez FJ (2015). TOLLIP, MUC5B, and the response to N-Acetylcysteine among individuals with idiopathic pulmonary fibrosis. Am J Respir Crit Care Med.

[8] https:/. /clinicaltrials.gov/study/NCT03312400 Last access 11 February 2024.

[9] Amati F, Spagnolo P, Ryerson CJ (2023). Walking the path of treatable traits in interstitial lung diseases. Respir Res.

[10] Amati F, Spagnolo P, Oldham JM (2023). Treatable traits in interstitial lung diseases: a call to action. Lancet Respir Med.

